# Longitudinal Course of Illness in Congenitally Deaf Patient with Auditory Verbal Hallucination

**DOI:** 10.1155/2022/7426850

**Published:** 2022-02-12

**Authors:** Yoshihiro Matsumoto, Nobutaka Ayani, Yurinosuke Kitabayashi, Jin Narumoto

**Affiliations:** ^1^Department of Psychiatry, Graduate School of Medical Science, Kyoto Prefectural University of Medicine, 465 Kajii-cho, Kawaramachi-Hirokoji, Kamigyo-ku, Kyoto 602-8566, Japan; ^2^Gojouyama Hospital, 4-6-3 Rokujounishi, Nara 630-8044, Japan; ^3^Department of Psychiatry, National Hospital Organization, Maizuru Medical Center, 2410 Yukinaga, Maizuru, Kyoto 625-8502, Japan

## Abstract

Auditory verbal hallucination is one of the core symptoms of schizophrenia, same as delusions, and also occurs in many other psychiatric disorders. Significant numbers of people with congenital deafness experience auditory verbal hallucinations; however, there are only a few reports regarding the course of psychosis in people with congenital deafness. Herein, we report the case of a patient with congenital deafness and auditory verbal hallucinations whose diagnosis was changed from psychotic major depression to schizophrenia 7 years after the onset of the disease. His psychotic symptoms decreased when his primary medication was changed from an antidepressant to antipsychotic drugs, based on the change of diagnosis. In the treatment of congenitally deaf patients with auditory verbal hallucinations, the inability to communicate through spoken language may interfere with proper diagnosis and treatment. The ability to collect detailed information in ways other than through verbal language is imperative for psychiatrists to determine the appropriate diagnosis and treatment for these patients during the longitudinal course of illness.

## 1. Introduction

Auditory verbal hallucination (AVH) is one of the core symptoms of schizophrenia, same as delusions, and also occurs in many other psychiatric disorders, such as major depressive disorder, bipolar disorder, and personality disorder [1]. To provide the appropriate treatment, it is important to review the diagnosis carefully throughout the course of the treatment [2]. Less than half of patients who experienced psychosis received a change of diagnosis in the last 10 years, and about 21–27% of patients with psychotic major depression (PMD) as a baseline diagnosis had their diagnosis changed to schizophrenia in the course of their lifetime diagnosis [2, 3].

Treatment of mentally ill patients with congenital deafness is more challenging compared to patients without hearing loss because of their inability to communicate through spoken language [4]. Congenital deafness occurs in approximately 1 to 3 of every 1,000 children [5], and these children may also experience AVH in the same way as people without deafness [6]. One study reported that the rate of AVH is about 50% among prelingually deaf patients with schizophrenia [6]. These results suggest that significant numbers of people with congenital deafness experience AVH, although the mechanism remains unclear [7]. Furthermore, to our knowledge, there are no reports regarding the longitudinal course of psychosis in people with congenital deafness.

In this paper, we report the case of a patient with congenital deafness and AVH whose diagnosis was changed from psychotic major depression to schizophrenia 7 years after the onset of the disease. His psychotic symptoms reduced when his primary medication was changed from an antidepressant to antipsychotic drugs, based on the reconsideration of the diagnosis.

## 2. Case Description

The patient was a 37-year-old man with congenital deafness. He had no history of harmful substance use or familial history of psychiatric disorders. He had a complication of ulcerative colitis; however, it was well-controlled for many years without the use of corticosteroids. His first psychiatric episode was of depression when he was 21 years old, which improved by administration of paroxetine. After that, he experienced several recurrences without psychosis because of difficulties in sustaining relationships or unemployment. When he was 29 years old, he experienced delusions, such as a strange woman sexually harassing him, with a depressive mood. When he was 30 years old, he experienced AVH with manifestations, such as someone saying, “mommy, mommy” or a TV announcer asking him, “what are you doing,” with a depressive mood. He was voluntarily admitted to a hospital for treatment, and he was diagnosed with PMD. His symptoms improved with the administration of 1 mg of risperidone and 37.5 mg of paroxetine controlled release and remained stable for 7 years with this medication regimen. When he was 37 years old, he again experienced depressive mood with AVH. The dosage of risperidone was increased to 1.5 mg, but it caused oversedation. His medication was changed from risperidone to 6 mg of aripiprazole, and his AVHs of “the mafia's wife asked him to become her friend” and “a mafia hit a desk hardly” became exacerbated while his mood improved. At that time, he expressed that he did not hear voices from his ears but rather felt the sound inside his head. Consequently, the dosage of aripiprazole was increased to 24 mg and paroxetine was tapered off gradually because his delusions and AVH were not consistent with his mood. Considering the psychotic symptoms continued without a depressive mood, the diagnosis was then changed from depression to schizophrenia. His psychosis improved with 6 mg of risperidone; however, 8 months later, he stopped taking it because of sexual dysfunction side effects, which caused his psychotic symptoms, including AVH, to recur and he was subsequently voluntarily admitted to our hospital. He had not used any psychoactive substances and there were no obvious abnormalities suggestive of organic or symptomatic factors on admission. The prescription was changed from risperidone to 15 mg of olanzapine, which was effective against the psychotic symptoms. One month later, he was discharged from the hospital. He continued to take12.5 mg of olanzapine for 2 years, without recurrence of his symptoms. The patient agreed to the publication of this report, and complete patient anonymity was ensured. The regulations of the Ethics Committee dictate that such clinical reports do not require committee approval. The communication with this patient was through a qualified interpreter of sign language and written conversation.

## 3. Discussion

This case report describes the longitudinal course of illness in a congenitally deaf patient with AVH and highlights two interesting points.

### 3.1. Diagnostic and Treatment Challenges in Patients with Congenital Deafness and Psychotic Symptoms

The patient in this case experienced psychosis including AVH. Although AVH is one of the core symptoms of schizophrenia, AVH alone does not confirm the diagnosis, as it is also frequently found in bipolar disorder (11–63%), borderline personality disorder (46%), or major depressive disorder (5–41%), and among the general population (10–20%) [1, 8]. In this case, the patient's therapists did not suspect that his course of illness was changing to schizophrenia until psychotic symptoms inconsistent with mood were observed, despite the fact that psychotic symptoms were frequently observed during the 7-year course of treatment. One of the most significant reasons that prevented the therapists from noticing the changes in this particular patient's course of illness was their inability to communicate with him through spoken language [4]. Therefore, communication with patients with congenital deafness should be conducted with a qualified interpreter of sign language and written conversation, in addition to careful history taking and behavioral observation. Epidemiology can also help to reassess a diagnosis when treatment responsiveness deteriorates. This patient was initially diagnosed with PMD, which has been reported to be liable to be changed, commonly to schizophrenia [9]. Among people without any hearing disability, one study reported that the diagnosis of PMD was changed to schizophrenia in 15/72 (20.8%) patients after10 years of follow-up [3]. Another study reported that the diagnosis of 15/55 (27.3%) patients with PMD was changed to schizophrenia [2]. Another implication in this case was that the patient stopped taking his medication because of sexual dysfunction side effects resulting in readmission. It is especially necessary when treating people with deafness who have difficulty communicating to carefully explain the side effects that may occur when starting a medication and provide strategies to cope with them before they occur.

### 3.2. AVH in People with Congenital Deafness

AVH is a phenomenon of hearing voices in the absence of any auditory stimuli. The inner speech theory is one of the major hypotheses for explaining the mechanism of AVH. This theory hypothesizes that inner speech has been misattributed to an external factor and is recognized as AVH [10]; this is a problem of impaired self-monitoring in patients. Pseudohallucinations and thought echo are similar phenomena to AVH. Pseudohallucinations also involve the phenomenon of hearing voices in the absence of any speaker; however, the patient can self-recognize it as a hallucination [11]. Thought echo, which is the patient hearing their own thoughts after thinking them, is one of Schneider's first rank symptoms of schizophrenia [12]. These phenomena could essentially be the same at the points of inner speech. The inner speech theory may explain AVH in people with congenital deafness; it explains why similar symptoms occur even though the inputs are different between people with and without deafness. In fact, the areas of the brain employed in language processing during inner speech are similar for people with and without deafness [13]. In contrast, Atkinson et al. [14] suggested that the perceptual characteristics of AVH relate closely to an individual's real-life communication preferences and experience of language. In this case, the patient reported AVH as inaudible in his ears but perceivable in his head, a description that is consistent with that given by Atkinson et al. The fact that this patient's expression of AVH differed from that of hearing people, but antipsychotic medications were effective in eliminating the AVH, may indicate that the etiology of AVH is similar in patients with and without deafness.

## 4. Conclusion

In the treatment of congenitally deaf patients with AVH, the inability to communicate through spoken language may interfere with proper diagnosis and treatment. In order to provide the appropriate treatment for these patients, it is imperative for psychiatrists to gather detailed information with the help of a qualified sign language and written language interpreter and to evaluate the validity of the diagnosis as appropriate during the longitudinal course of the patient's illness.

## Data Availability

The data used to support the findings of this study are available from the corresponding author upon request.
